# Nucleation of glucose isomerase protein crystals in a nonclassical disguise: The role of crystalline precursors

**DOI:** 10.1073/pnas.2108674119

**Published:** 2022-01-31

**Authors:** Alexander E. S. Van Driessche, Wai Li Ling, Guy Schoehn, Mike Sleutel

**Affiliations:** ^a^Université of Grenoble Alpes, CNRS, ISTerre Grenoble F-38000, France;; ^b^Instituto Andaluz de Ciencias de la Tierra (IACT), CSIC-University of Granada, 18100 Armilla, Granada, Spain;; ^c^Université Grenoble Alpes, CEA, CNRS, IBS Grenoble F-38000, France;; ^d^Department of Bioengineering Sciences, Structural Biology Brussels, Vrije Universiteit Brussel 1050 Brussels, Belgium;; ^e^Structural and Molecular Microbiology, VIB-VUB Center for Structural Biology, VIB 6 1050 Brussels, Belgium

**Keywords:** nucleation, proteins, self-assembly, crystallization, precursor phase

## Abstract

Protein crystallization is an astounding feat of nature. Even though proteins are large, anisotropic molecules with complex, heterogeneous surfaces, they can spontaneously group into two- and three-dimensional arrays with high precision. And yet, the biggest hurdle in this assembly process, the formation of a nucleus, is still poorly understood. In recent years, the two-step nucleation model has emerged as the consensus on the subject, but it still awaits extensive experimental verification. Here, we set out to reconstruct the nucleation pathway of the candidate protein glucose isomerase (GI), for which there have been indications that it may follow a two-step nucleation pathway under certain conditions. We find that the precursor phase present during the early stages of the reaction process is nanoscopic crystallites that have lattice symmetry equivalent to the mature crystals found at the end of a crystallization experiment. Our observations underscore the need for experimental data at a lattice-resolving resolution on other proteins so that a general picture of protein crystal nucleation can be formed.

## Significance

The ability of proteins to self-assemble into complex, hierarchical structures has been the inspiration for the bottom-up design of a class of biomaterials with proteins as their building blocks. The earliest stages of formation often involve the passing of an activation barrier under the form of nucleus formation, a quaternary protein complex that templates incoming molecules to proper registry. For protein crystallization, the consensus has emerged that the fastest route toward a nucleus follows a winding path: first, densification, followed by symmetry formation. In this contribution, we show that this need not be the case for the protein glucose isomerase, which seems to follow the simplest path to a nucleus, making crystalline clusters from the earliest detectable beginnings.

Th e formation of a new phase within a supersaturated ambient phase starts out with the creation of a tiny embryonic seed that is called the nucleus. The nucleus acts as a template for further growth at the expense of the ambient phase, until equilibrium is reached. In the simplest model of nucleation that one can imagine, the nanoscopic seed is structurally identical to the macroscopic version of the new phase found at equilibrium. This structural equivalence between the nucleus and the bulk is the basic principle of the theoretical treatment of nucleation developed by Gibbs in the late 19th century (1, 2), which lay the foundation of the classical nucleation theory (CNT) (3) that is still being used today. Attractive by its conceptual simplicity and ability to semiquantitatively reproduce the nucleation kinetics of a broad range of systems, CNT and its subsequent adaptations had become the most widely used theory on nucleation. However, that status was challenged when technical advancements paved the way for experiments and simulations to directly follow the process of crystal nucleation at molecular and even atomic resolution. This has led to a virtual barrage of publications reporting on nucleation pathways that do not fit the predictions made by CNT and in doing so triggering a renaissance of the nucleation topic (4–11).

In this contribution, we focus on the crystallization of the protein glucose isomerase (GI). Proteins have become strongly associated with an alternative model of nucleation [i.e., two-step nucleation (12)]. In its most general form, the two-step model assumes that protein molecules first self-assemble into a disordered, mesoscopic “droplet,” which later reorganizes its internal state to develop crystalline symmetry. This process is thought to involve the passing of two separate activation barriers, which has led to the adoption of the term two-step nucleation. This two-step concept was initially formulated by ten Wolde and Frenkel (13) based on numerical simulations, and it has since been (in)directly supported by experimental evidence (5, 14–18) for various colloid and protein systems. The most convincing support for two-step nucleation for proteins comes from recent cryogenic transmission electron microscopy tomography observations on ferritin by Houben and coworkers (5), who reported an increase in both order and density from the surface of early formed ferritin aggregates toward their interior, gradually transforming into crystalline nuclei. That densification and emergence of order seem to occur in tandem for ferritin, which is distinct from the two-step model where both steps are assumed to be decoupled. Moreover, it remains unclear if this nucleation pathway for ferritin involves two separate nucleation events—one associated with the initial aggregate formation and one with the condensation and crystallization step of said aggregates—or if this should be viewed as a cooperative process characterized by a single activation barrier. For a broader perspective on multistep and nonclassical nucleation, we refer to the following works (19–21) on proteins, as well as small molecules and (in)organic salts.

For now, ferritin is the only example where molecular-resolution information on a two-step nucleation-like process is available. It is therefore not possible to know if the observed discrepancies between the two-step model and the experimental ferritin pathway should serve as a source of corrections for future theoretical treatments or if ferritin is an outlier on the two-step theme? To make that distinction, more experimental data are required for other protein-precipitant systems. For that, we work with GI, whose mechanism of nucleation has been suggested to follow a two-step pathway mediated by prenucleation submicron particles whose composition and internal structure remain unknown (22). We set out to experimentally characterize these ill-defined prenucleation species [that emerge before the formation of the final crystalline phase and which have been observed for a host of other systems (16, 22–28)] whose presence in the mother liquor accelerates the rate of nucleation. Interestingly, GI exhibits polymorphism between two different orthorhombic space groups (I222 and P2_1_2_1_2) (29, 30), and the two-step nucleation features have been solely attributed to the I222 polymorph (22). Moreover, it is clear that the nucleation pathway of the P2_1_2_1_2 space group does not entail a liquid-like precursor phase but rather involves hierarchical self-assembly of nanocrystalline rods (4). The potential role of a disordered precursor phase on the nucleation of I222 crystals is the subject of this study.

Here, we revisit the nucleation of I222 GI crystals and study nonfiltered protein stock solutions by means of cryogenic electron microscopy (cryoEM). We demonstrate that the alleged prenucleation particles are in fact I222 nanocrystals that are formed because of high supersaturation that momentarily occurs during the preparation of the protein stock solution. This observation calls into question the precise nature of other prenucleation particles that have become nearly synonymous with two-step nucleation, as well as the generality of the two-step nucleation mechanism.

## Results

### Submicron Particles Enhance the I222 Nucleation Rate of GI.

**Fig. 1.**
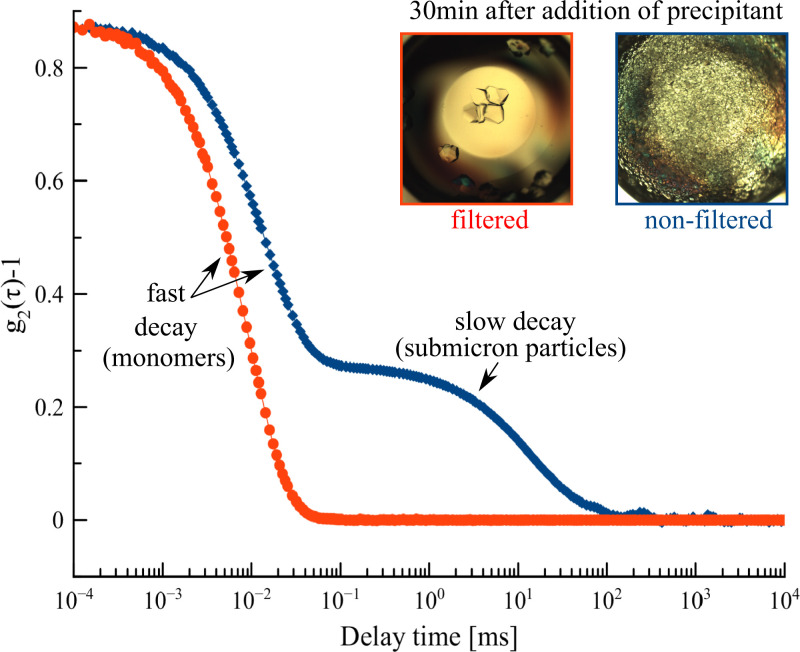
Intensity correlation function of a filtered (red) and nonfiltered (blue) GI solution: 173 mg ⋅ mL^−1^, 10 mM Hepes pH 7.0, 1mM MgCl_2_; *Top Inset*: photographs of both solutions taken 30 min after mixing with 100 mM Hepes pH 7.0, 200mM MgCl_2_, 8% (wt/vol) PEG 1000.

We have previously established that submicron particles (100 nm to 1 µm) can form in highly concentrated (± 200 mg ⋅ mL^−1^) GI solutions with low ionic strength (e.g., 1 mM MgCl_2,_ 10 mM Hepes) (22). When monitored using dynamic light scattering (DLS), such unfiltered GI solutions exhibit a secondary decay in their intensity correlation function that corresponds to a population of slowly diffusing particles (Fig. 1). These particles remained stable in solution at 4 °C over extended periods (>months). Throughout the storage period at 4 °C, we did not detect any crystalline objects within these solutions using conventional polarized light microscopy. We can, however, readily induce crystallization by adding poly-ethylene glycol (PEG) or ammonium sulfate (AS) to the protein solution. For example, if we mix equal volumes of 173 mg ⋅ mL^−1^ GI, 10 mM Hepes 7.0, 1 mM MgCl_2_ with 100mM Hepes pH 7.0, 200mM MgCl_2_, 8% (wt/vol) PEG 1000, then orthorhombic I222 crystals become visible within minutes (Fig. 1). The number of crystals that we observe after 30 min depends on whether we filter the protein stock solution prior to mixing with PEG 1000 (Fig. 1). The filtered solutions do not give rise to crystal formation within 30 min after mixing with the precipitant, whereas the unfiltered solutions do. Moreover, they do so in a manner that is proportional to the GI concentration [i.e., conditions with higher protein concentration yield more crystals and vice versa (*SI Appendix*, Fig. S1)]. Close examination using polarized microscopy of the mother liquor solutions that were prepared using unfiltered GI, shows that crystals are already formed within the dead time of the experiment (1 min). This means that the induction time for crystal formation for the unfiltered samples is close to zero. The mother liquors that were prepared with filtered GI, however, only developed crystals hours after mixing with PEG 1000 and therefore have a nonzero induction time.

A vanishingly small induction time is a feature that is shared between systems that either approach spinodal decomposition or systems that are dominated by heterogeneous nucleation on preexisting surfaces. We can rule out the former by lowering the protein concentration and with it the supersaturation (*SI Appendix*, Fig. S1): we observe that the crystal number density scales with the supersaturation, but the induction time remains smaller than the experimental dead time. From this we conclude that the rate of homogenous nucleation is negligible in comparison to the rate of heterogeneous nucleation. Noteworthy, this triggering of crystal nucleation is polymorph specific; only I222 crystals are induced even though we are working in a regime where GI exhibits polymorphism between the space groups I222 and P2_1_2_1_2. These results suggest that the submicron particles serve as crystal nucleation centers in a polymorph-specific manner, but they provide no further insight into the mechanism behind this seeding process.

### The Alleged Mesoscopic Prenucleation GI Particles Are Submicron I222 Crystals.

**Fig. 2.**
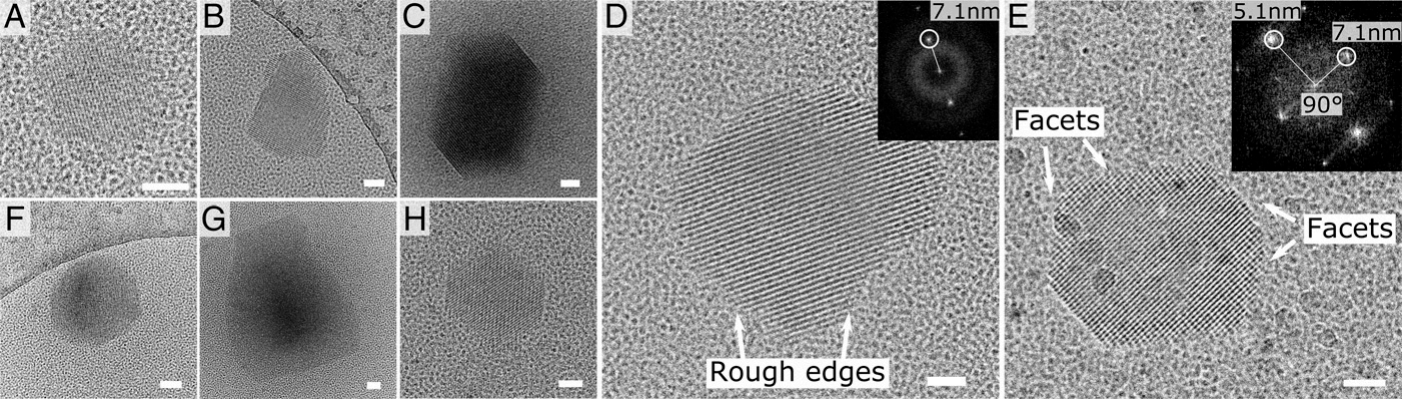
Crystalline GI clusters in concentrated stock solutions of GI: 173 mg ⋅ mL^−1^ GI, 10 mM Hepes pH 7.0 and 1 mM MgCl_2_ (*A–E*), 2 mM MgCl_2_ (*F*), or 1 mM MgCl_2_ with 4% (wt/vol) PEG 1000 (*G* and *H*). In panel (*E*), we highlight the rough and straight facets of the nanocrystal and the corresponding fringes along two directions. The power spectrum of the nanocrystal (*Inset*) exhibits maxima that correspond to the experimentally measured distances between the lattice planes of the I222 space group. All scale bars represent 50 nm. Images (*A–F*) were collected from GI solutions that were prepared 48 h prior. The images (*G* and *H*) were collected minutes after the mixing of the protein and precipitant solutions.

To gain further insight into the role that these preformed GI particles play in the nucleation process of I222 crystals, we employed cryoEM on plunge-frozen aliquots of an unfiltered GI stock solution (173 mg ⋅ mL^−1^ GI, 1 mM MgCl_2_) without any PEG 1000 or AS present to increase the supersaturation. A total of 3 µL of the stock solution was applied to a cryoEM grid, after which excess solution was blotted away. The resulting thin liquid film suspended over the holes of the cryoEM grid was vitrified by rapidly plunging the grid into liquid ethane cooled by liquid nitrogen at a rate of 10^4^ K/s, inhibiting any further crystallization during the grid preparation process. These grids were subsequently preserved and imaged at liquid nitrogen temperature. By carefully screening the grids, we could detect the presence of (partially) facetted submicron particles that measure 280 ± 166 nm (*n* = 12; SD) (Fig. 2 *A–C* and *SI Appendix*, Fig. S2) at 1mM MgCl_2_. The absence of fully developed facets for some of these nanocrystals (Fig. 2 *A*, *B*, *D*, *F*, and *G*) may follow from two different mechanisms: 1) their formation process may involve a noncrystalline precursor species, which upon crystallizing gave rise to some of the erratic crystal habits that were detected or 2) they were formed at high supersaturation levels and therefore deviate significantly from the expected Wulff shape due to kinetic roughening (31). As we did not manage to record any images of GI states that precede the formation of the observed nanocrystals, we cannot make this distinction between the two scenarios.

Also, it should be noted that our sample preparation protocol may introduce a bias toward smaller crystallites, as larger crystals may have been removed during the blotting process of the EM grid prior to the plunging into liquid ethane. In a recent study, Tsarfati et al. (21) obtained qualitatively similar results for grids prepared with and without blotting, but they showed that blotless grid preparation can indeed lead to the trapping of larger particles. Here, we obtain similar values for the crystal dimensions at 2 mM (270 ± 66 nm; *n* = 8; SD) and 4 mM MgCl_2_ (205 ± 62 nm; *n* = 25; SD). However, if we supplement the 1 mM MgCl_2_ GI solution with PEG 1000 to a final concentration of 5% (wt/vol) immediately before deposition onto a grid, then we record a marked increase in the crystal dimensions (590 ± 290 nm; *n* = 21; SD). This rapid growth is in line with our earlier crystallization trials, which showed that addition of PEG 1000 leads to rapid formation of macroscopic I222 crystals following the drastic reduction of the GI solubility (32). This suggests that the addition of PEG 1000 triggers the rapid growth toward macroscopic crystals of the nanocrystallites that were already present in the protein stock solution.

When oriented properly, the nanocrystals exhibit lattice fringes in the cryoEM micrographs, and the resulting maxima in the power spectrum (e.g., Fig. 2*E*) correspond to the intermolecular distances between the lattice planes. The measurements here (5.1 and 7.1 nm at a 90° angle) are in line with previous observations for I222 GI crystals and fit predictions based on X-ray crystallographic measurements of macroscopic crystals (4). We therefore conclude that these crystals are nanoscopic renditions of the mature I222 crystals that we obtain in our crystallization experiments and that they can be selectively removed via filtration (*SI Appendix*, Fig. S3). This readily explains the seeding behavior at the macroscopic level, as well as the specificity regarding the I222 polymorph. However, it also raises certain questions about their origin, stability, and submicron size.

**Fig. 3.**
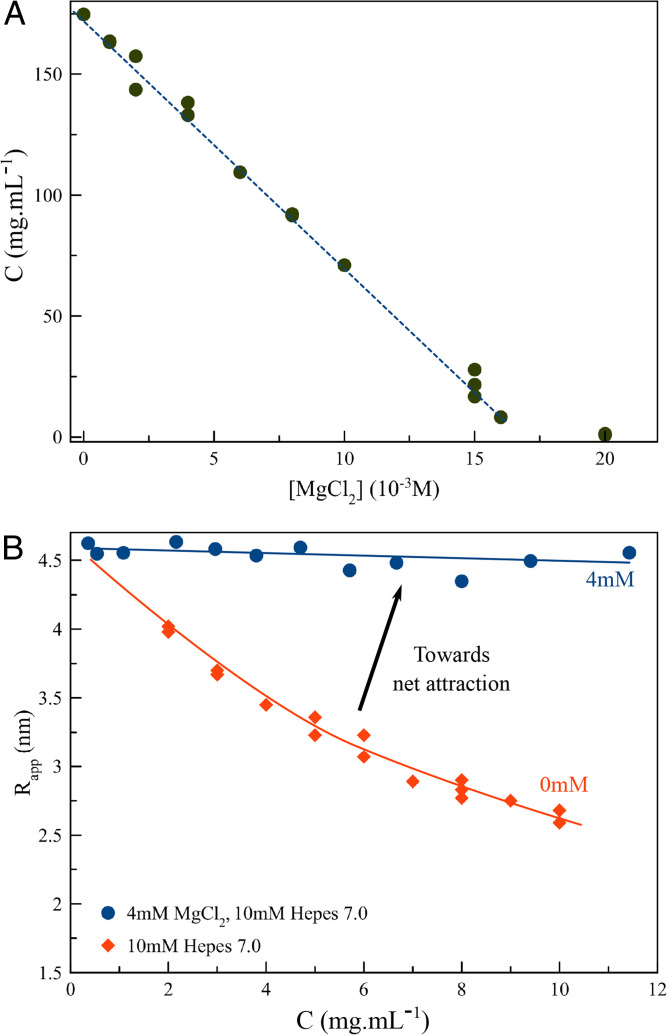
(*A*) Solubility curve of orthorhombic GI crystals as a function of MgCl_2_ concentration; (*B*) Apparent hydrodynamic radius of GI determined using DLS, as a function of protein concentration at 0 and 4 mM MgCl_2_. Lines are guides for the eye.

We first address their stability by determining if the GI stock solution is in equilibrium or supersaturated with respect to the I222 phase. For this, we determined the crystal–liquid coexistence curve as a function of the concentration of MgCl_2_ (Fig. 3*A*). Typically, MgCl_2_ is added to GI in low millimolar concentrations because the active site holds two metal cofactors (33) (e.g., Co, Mn, and Mg) that contribute to the thermal stability of the protein (34), but this also affects the solubility (30). The GI equilibrium concentration for the conditions used here is highly sensitive to the MgCl_2_ concentration as it decreases 500-fold from 1 to 20 mM MgCl_2_. We note that there is no salting-in regime, a common feature in the low-salt region of the phase diagram of many proteins; instead, GI only exhibits salting-out over the tested range. Such high sensitivity to MgCl_2_ could indicate that Mg^2+^ interacts in a specific manner with the surface of GI, or indeed in the formation of salt bridges in lattice contacts, as has been the case for other protein crystals [e.g., insulin (35), Zn^2+^, PDB: 1G7A; ferritin (36)], Cd^2+^, PDB: 1LB3). To this end, we collected an in-house X-ray dataset on I222 crystals grown in 20 mM MgCl_2_ but found no meaningful structural differences with the lattice contact of I222 crystals grown in 0.8 M AS with no Mg^2+^ present (*SI Appendix*, Fig. S4). Moreover, crystallization is not specific to Mg^2+^ since we obtain qualitatively similar results with other divalent cations, such as Ca^2+^, Zn^2+^, Ni^2+^, Cu^2+^, and Mn^2+^, which all trigger I222 nucleation in the 2.5- to 50-mM range. In fact, monovalent cations can also be used as a salting-out agent, albeit at slightly higher concentrations (e.g., 100 mM NaCl).

It therefore seems likely that the cations we tested facilitate macromolecular self-assembly by shielding the electrostatic repulsion between GI molecules in a nonspecific manner (at pH 7.0, GI has a net negative charge of −74e; https://www.protpi.ch/). We attribute that large negative charge to the absence of a salting-in regime in the MgCl_2_ solubility curve of GI (Fig. 3*A*). The salting-out effect by MgCl_2_ is reflected in the apparent hydrodynamic radius (*R*_app_) of the monomers measured by DLS (Fig. 3*B*). In 10 mM Hepes 7.0, *R*_app_ decreases monotonically as a function of the GI concentration. The slope (*k*_D_^−1^) of this curve (Fig. 3*B*) is a measure of the pairwise interaction potential between the GI molecules averaged over all possible orientations (37). The negative sign of *k*_D_^−1^ indicates net repulsion between the solute molecules. The value of *k*_D_^−1^ approaches zero for the curve collected at 4 mM MgCl_2_, implying that the repulsive forces between the particles are greatly diminished due to electrostatic shielding, which is in line with the general trend of the solubility curve.

Our solubility measurements (Fig. 3*A*) also demonstrate that the crystals shown in Fig. 2 are not in equilibrium with their surrounding liquid: the bulk GI concentration of our protein stock solution was 173 mg ⋅ mL^−1^, which is 6% higher than the equilibrium concentration recorded for this condition (*C*_e_ is 163 mg ⋅ mL^−1^ at 1 mM MgCl_2_). Since the protein stock solution is supersaturated, we expect that the nanocrystals should still be able to grow until equilibrium is reached. Such an equilibrium may not necessarily be reached in a practical timeline given that the kinetics could be very slow because of kinetic barriers that trap these nanocrystals in a metastable state. Clues to the origins of such barriers can be found in the crystal morphology. Some crystal facets are straight with sharp corners at the edges, suggesting full completion of the outer layers (e.g., Fig. 2 *D* and *E*). From this, we conclude that crystals with fully developed habits are of (near) magic size. Magic nuclei are metastable toward further expansion because new molecular layers need to be initiated on the existing facets for growth to continue. It is the work associated with the formation of a new molecular island that gives rise to metastability. We can estimate the impact on the kinetics of crystal growth by predicting the two-dimensional nucleation rate using kinetic parameters that we determined previously (31, 32). For a supersaturation of ln(173/163) = 0.059, we predict a nucleation rate of two-dimensional islands to be 1.2 × 10^6^ m^−2^ ⋅ s^−1^. Assuming nucleation to be the rate-limiting step, this translates roughly into the deposition of one new molecular layer per 60 d. Such a slow growth rate practically means that the fully facetted nanocrystals are kinetically frozen, and the partially facetted nanocrystals are expected to grow further until their facets are completed thus halting further growth.

**Fig. 4.**
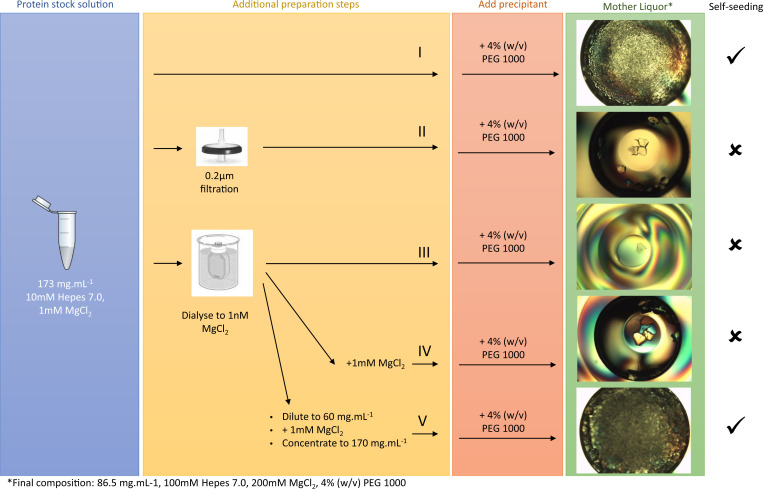
Reversibility of the self-seeding effect upon removal and subsequent addition of MgCl_2_ during the concentrating step: the concentrated protein stock solution is either used as is and mixed with PEG 1000 (I), or first passed through a 0.2 µm syringe filter (II), or dialyzed twice against 10 mM Hepes pH 7.0 to lower the [MgCl_2_] to 1 nM but keeping the protein concentration constant (III), after which we resupplement MgCl_2_ to bring it to 1 mM final (IV) or dilute first in 10 mM Hepes 7.0 to bring the protein concentration to 60 mg · mL^−1^, resupplement MgCl_2_ to 1 mM final and reconcentrate glucose isomerase to 170 mg · mL^−1^ prior to mixing with PEG 1000 (V).

We also note that the induction time for crystal formation is inversely proportional to the supersaturation. For instance, at 2 mM MgCl_2_ with no other precipitant present, we start to observe macroscopic crystals after an incubation time of months, whereas at 20 mM, crystals become detectable as soon as 24 h. Conversely, if we lower the MgCl_2_ concentration by dialyzing the 1-mM condition to 10 mM Hepes 7.0 (expected final MgCl_2_ concentration after dialysis of 1 mL GI against 2 × 1l is 1 nM), then the seeding effect upon mixing with 8% (wt/vol) PEG 1000 is lost [i.e., we see a drastic reduction (III in Fig. 4) of the final crystal count toward the level we observe for a filtered solution (II in Fig. 4)]. This suggests that the nanocrystals have dissolved because of the undersaturation that was imposed. If we resupplement the dialyzed solution with MgCl_2_ to a final concentration of 1 mM (IV in Fig. 4), then we do not see an increase in the final crystal count, which means that nanocrystals likely not reformed due to the low supersaturation (0.059) in this condition.

This brings us to the final conundrum: why did nanocrystals form in the protein stock solution (173 mg ⋅ mL^−1^ GI, 1 mM MgCl_2_) in the first place? The answer can be found in the last preparation step of the protein stock solution, where we reconcentrate the dialyzed GI after diluting to 60 mg ⋅ mL^−1^ (i.e., undersaturated) via a spin concentrator (V in Fig. 4). We found that there is a noticeable build-up of GI at the bottom of the concentrator after 10 min of centrifugation at 7,500 relative centrifugal field (rcf)—such local gradients are clearly visible due to the yellow/orange hue of GI at high concentrations. Indeed, we recorded GI concentrations in ex situ aliquots, taken from the liquid region in closest contact to the filter of the concentrator, that are in the order of 300 mg ⋅ mL^−1^, but higher local concentrations (and therefore supersaturation) likely exist in situ as well. Note that these gradients are neutralized at the end of the concentrating step when the entire volume is transferred to a new Eppendorf and mixed anew. If we resupplement MgCl_2_ to the 1-nM solution that was obtained after dialysis, then we indeed recover a drastic increase in the number of macroscopic crystals obtained after concentrating and mixing with PEG (V in Fig. 4). This demonstrates full reversibility of the process and is in line with classical nucleation of GI nanocrystals in the local areas of high supersaturation.

We conclude that GI nanocrystals have unexpectedly formed in our protein stock solutions because of a short but deep quench below the binodal—a quench that is likely to occur for other proteins as concentrating via centrifugal filters is a common practice for any protein crystallization experiment. The nuclei that were formed during this brief period retained their submicron size due to kinetic barriers during subsequent (long-term) storage at weak driving forces, essentially serving as a stabilized seed stock awaiting a supersaturation trigger to continue growth. Our efforts to characterize the formation process of the I222 nanocrystals were ultimately unsuccessful, as we were not able to observe any GI structures that were smaller than the crystals reported in Fig. 2. To make definitive statements on the precise nature of the I222 nucleation process, and whether the precursor species are ordered or disordered in nature, further study is required.

## Discussion

By now it is well established that submicron-sized particles can spontaneously develop in concentrated protein solutions before the emergence of a crystalline state (16, 22–28). Indeed, particles with radii ranging from tens to hundreds of nanometers have been detected in solutions of lysozyme (38), GI (22), canavalin (27), lumazine synthase (24), hemoglobin (39), and others. We note that these particles are distinct from liquid dense clusters (40, 41) that arise in the formation process of membraneless protein microcompartments that are associated with neurodegenerative diseases, gene regulation, signaling, etc. These membraneless organelles are reversibly formed in vivo through a process of liquid–liquid phase separation.

Conversely, here we are referring to protein aggregate structures that spontaneously develop in high-density solutions of otherwise soluble proteins. The relevant feature of the particles that we refer to is their stability in size (i.e., they remain mesoscopic [submicron] over extended periods of time [months]). This steadiness suggests a stabilization mechanism that limits further growth of these particles to reach macroscopic sizes (42–44). The mesoscopic nature differentiates them from classical protein aggregation driven by nonspecific hydrophobic and/or electrostatic interactions yielding particulates in the µm to mm range (45). Secondly, these mesoscopic particles have been shown to actively contribute to the growth of protein crystals (46). They can do so by merging with the parental lattice and triggering the formation of new molecular layers that than serve as molecular addition sites. That ability to flawlessly merge with crystalline surfaces has been the cornerstone of the hypothesis that these particles are structurally plastic, or even liquid like—the rationale being that solid or even crystalline objects would not be able to merge without the introduction of severe lattice mismatches. However, that argument has been invalidated by recent observations of protein nanocrystals undergoing oriented attachment (47) into a mutual, unified lattice (*SI Appendix*, Fig. S5). And thirdly, it has been shown that these particles can actively participate in the nucleation process by serving as the centers of birth of new crystals, reminiscent of the process of secondary nucleation. That observation reinforced the notion that these particles could take up the role of the metastable precursor phase in the two-step nucleation model (22).

The questions formulated in the Introduction have remained largely unanswered for most proteins and called for additional in situ data. Our cryoEM observations for GI now allow us to formulate a different model for particle-mediated nucleation without invoking a two-step nucleation scenario. Rather, our data shows that the submicron particles are I222 GI nanocrystals that are formed during a protein concentrating step before the addition of precipitant. This leads to the installment of a region of high supersaturation close to the filter, which temporarily (for the duration of the concentrating step) triggers the nucleation of I222 crystals. This readily explains the I222-specific seeding effect and their size stability: nucleation and growth only occur during a short window and become negligible during long-term storage because of the very low (but positive) supersaturated state.

These data show that in the absence of high-resolution in situ data, it is difficult to make definitive statements regarding the structural nature of precursor states that precede the emergence of macroscopic protein crystals. Although our results are limited to GI, it does raise questions regarding the suggested ubiquity of the two-step nucleation scenario for proteins and calls for additional data for other systems.

## Materials and Methods

### GI Crystallization.

GI was received as a crystalline slurry from Macrocrystal Oy. Preparation of soluble GI stocks was done as follows: 5 mL GI crystal suspension was dialyzed (SpectraPor dialysis tubing, 10-kDa molecular weight cut off [MWCO]) against 2 × 2l 10 mM Hepes 7.0, 1 mM MgCl_2_. The solubilized GI solution was passed through a 0.2-µm syringe cutoff filter (Thermo Scientific) to remove any particulates and subsequently concentrated using a 100-kDa MWCO centrifugal filter (Amicon). The final concentration of the protein stock solution was determined via absorption at 280 nm using the extinction coefficient 1.042 mg^−1^ ⋅ mL ⋅ cm^−1^. To initiate crystallization, the GI stock solution was diluted to the desired concentration using 10 mM Hepes 7.0, 1 mM MgCl_2_ and mixed in a 1:1 (vol/vol) ratio with 100 mM Hepes pH 7.0, 200mM MgCl_2_, 8 to 11% (wt/vol) PEG 1000 at room temperature.

### Cryogenic Electron Transmission Microscopy.

A total of 3 µL of the sample was applied to freshly glow discharged qauntifoil or lacey carbon film grid and vitrified using a Thermofisher Vitrobot. Images were acquired with a Ceta CMOS camera under low-dose conditions on a Tecnai F20 microscope operating at 200 keV or with a Gatan K2 direct electron detector on a Polara microscope at 300 keV.

### DLS.

Intensity correlation functions of filtered and nonfiltered GI stock solutions were collected at 20 °C in 4-µL disposable cuvettes using a DynaPro Nanostar (Wyatt).

### Solubility Measurements.

Pregrown I222 GI crystals were centrifuged (5 min, 20,000 rcf) and washed three consecutive times using 1-mL fractions of 10 mM Hepes 7.0 and 0 to 20 mM MgCl_2_ before being stored at 4 °C. Protein concentration determination of the soluble phase after centrifugation was done on a weekly basis until a steady state was reached.

## Supplementary Material

Supplementary File

## Data Availability

All study data are included in the article and/or *SI Appendix*.
