# Supplementary material for: Nucleation of glucose isomerase protein crystals in a nonclassical disguise: The role of crystalline precursors
Source: Proc Natl Acad Sci U S A. 2022 Jan 31;119(7):e2108674119. doi: 10.1073/pnas.2108674119 (PMC8851477; doi:10.1073/pnas.2108674119)
Supplement: Supplementary File [file pnas.2108674119.sapp.pdf]

**Supporting Information to**

Nucleation of glucose isomerase protein crystals in a non-classical disguise: the role of crystalline precursors

**Authors**: Alexander E.S. Van Driessche[1], Winnie Ling[2], Guy Schoehn[2], and Mike Sleutel[3,4*]

**Affiliations:**

[1] Univ. Grenoble Alpes, CNRS, ISTerre, F-38000 Grenoble, France

[2] Institut de Biologie Structurale, IBS/Methods and Electron Microscopy Group, 41 rue Jules Horowitz, 38027 Grenoble Cedex 1 - France

[3] Structural Biology Brussels, Vrije Universiteit Brussel, Pleinlaan 2, 1050 Brussels, Belgium

[4] Structural and Molecular Microbiology, Structural Biology Research Center, VIB, Pleinlaan 2, 1050 Brussels, Belgium

[*]Correspondence to: mike.sleutel@vub.be

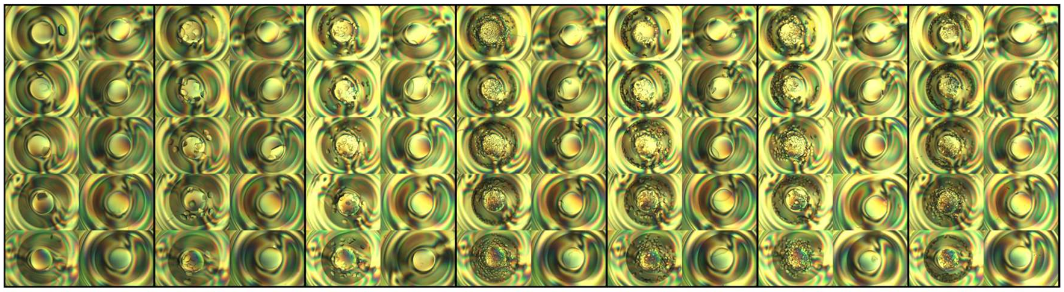


Supporting Figure 1: GI crystallization experiments as a function of GI concentration (10-70 mg/ml). Columns labelled as 'unfilt' and 'filt' represent mother liquor solutions that were prepared by mixing equal volumes of the 0.22µm filtered or unfiltered, respectively, protein stock solutions and a twofold concentrated precipitant solution.

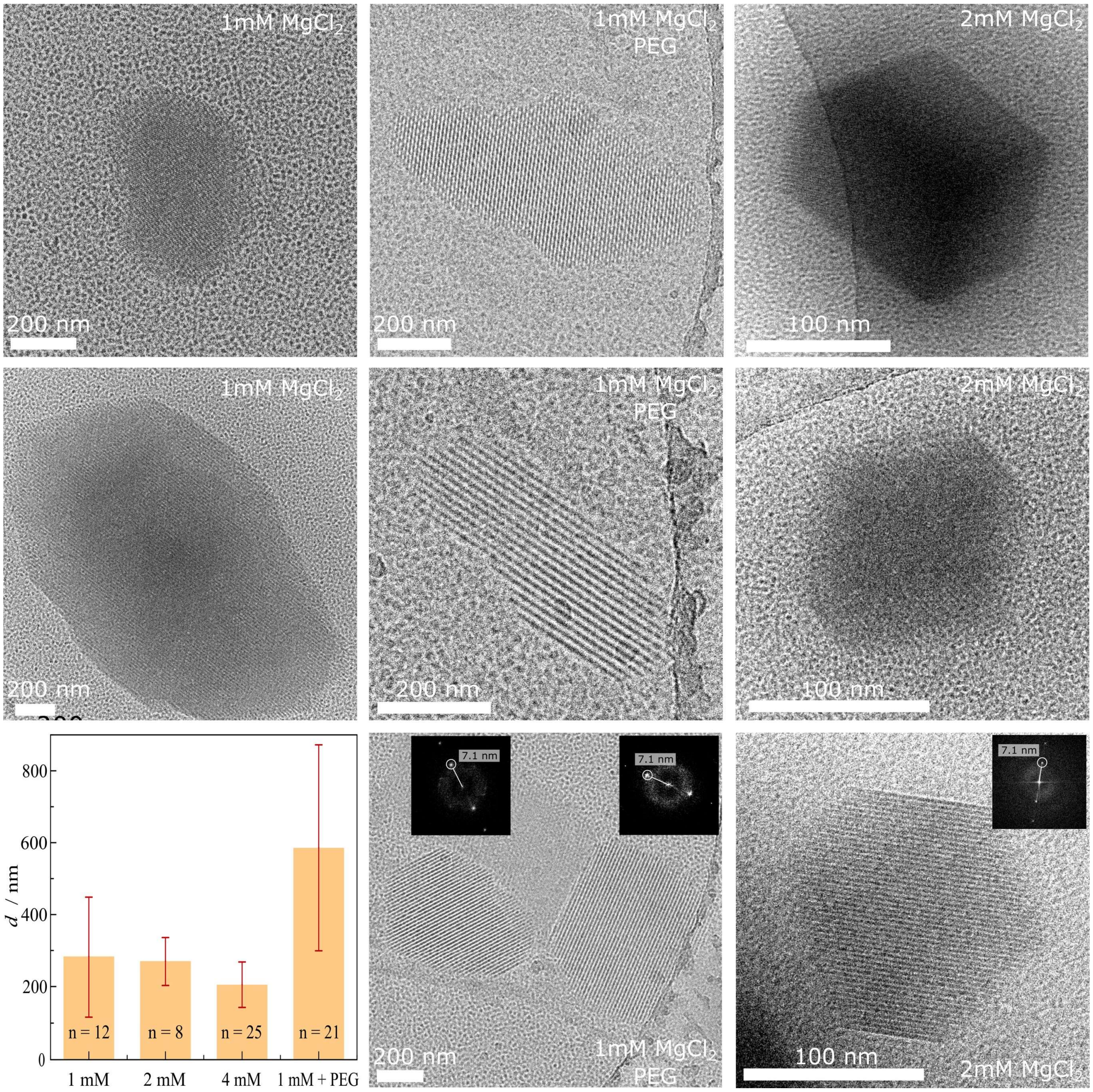


Supporting Figure 2: GI I222 nanocrystals imaged by cryoEM at 10mM Hepes 7.0 and 1mM, 2mM or 4mM $MgCl_2$, or 1mM $MgCl_2$ with 5% (w/v) PEG 1000, and the corresponding dimensions, with n the number of measured objects.

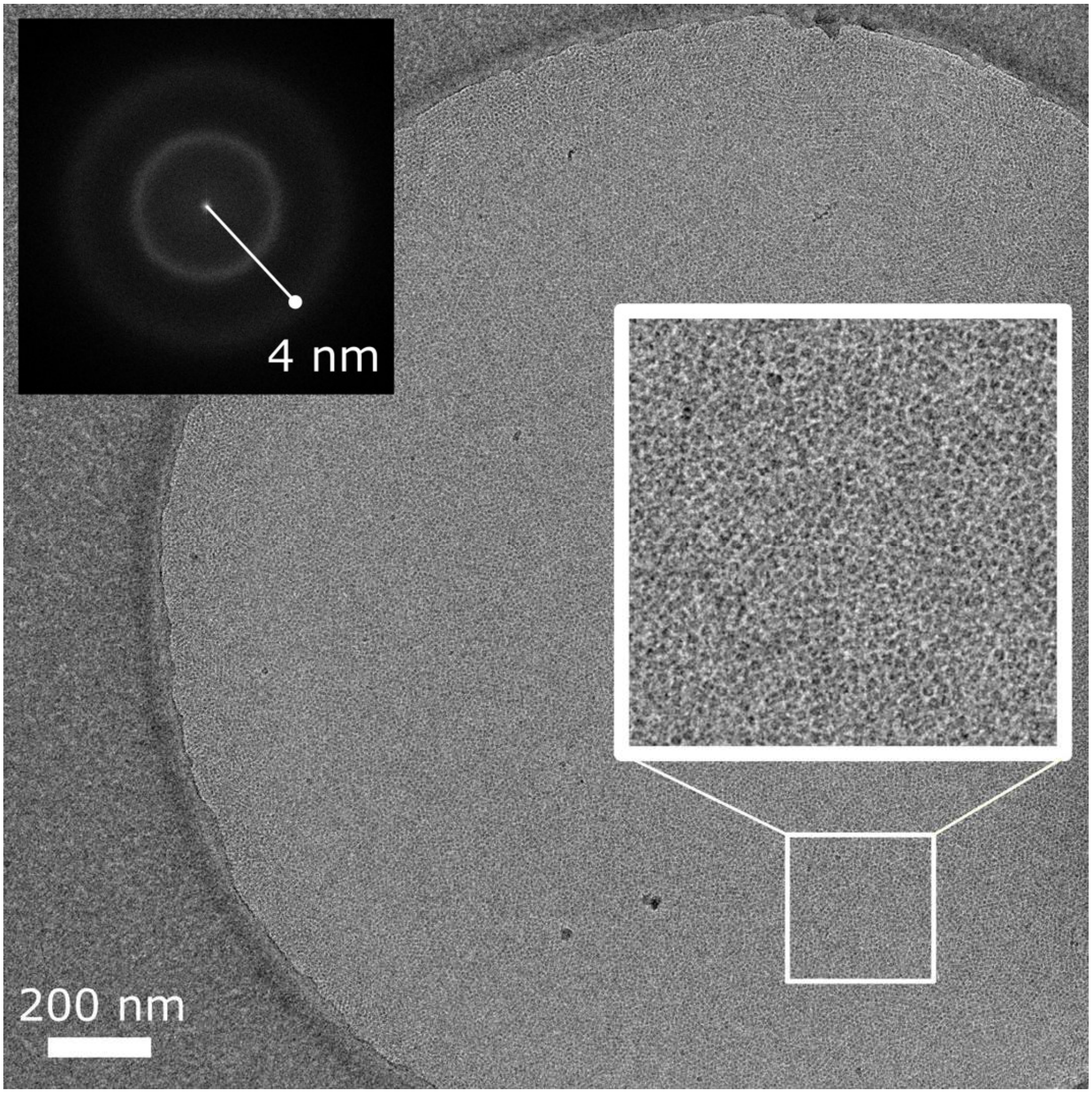


Supporting Figure 3: CryoEM image of a 173 $mg.mL^{-1}$ GI, 1mM $MgCl_2$ GI stock solution filtered with a 0.2µm cutoff syringe filter 24h before plunging. No crystallites or GI aggregates were detected.

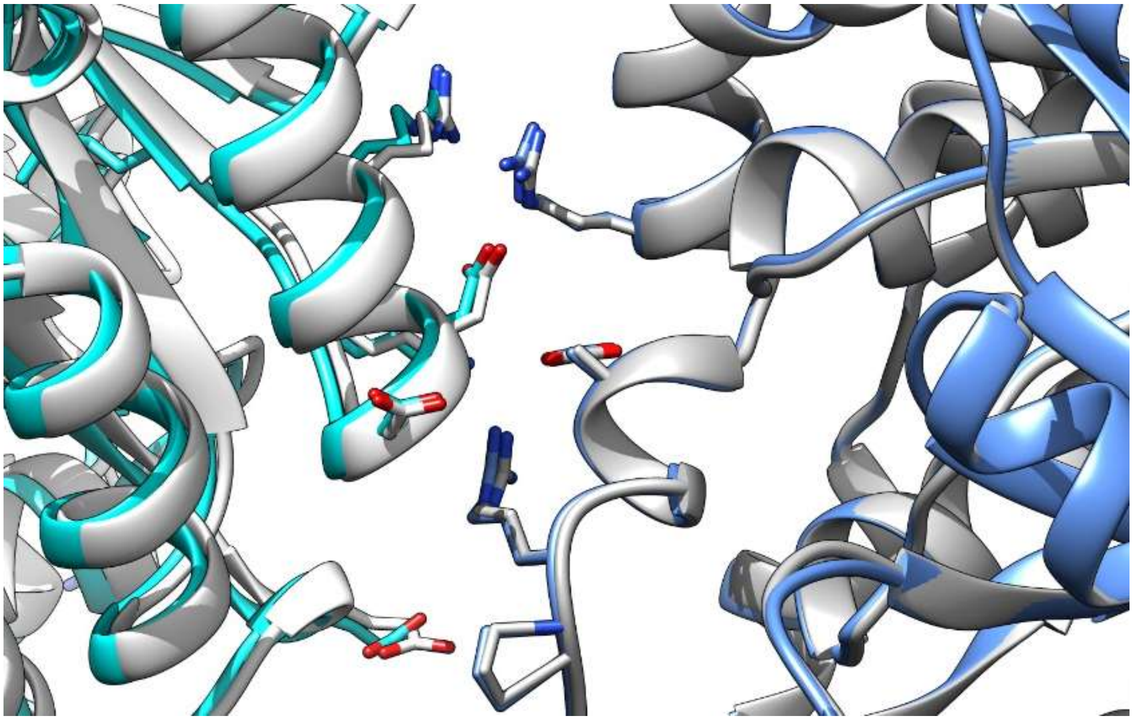

Supporting Figure 4: Comparison of the lattice contact for the I222 space group grown without $Mg^{2+}$ (symmetry related molecules shown in white and grey) and with $Mg^{2+}$ (cyan and blue). The Mg-less contact is based on the GI structure with PDB id 9XIA, which was crystallized in 10mM PIPES 7.4, 0.76M $(NH4)_2SO_4$ and 25 mg.mL$^{-1}$ glucose isomerase, whereas the latter is based on a GI structure that was determined in-house by diffracting crystals that were grown in 10mM Hepes 7.0, 20mM $MgCl_2$ and 30 mg.mL$^{-1}$ glucose isomerase.

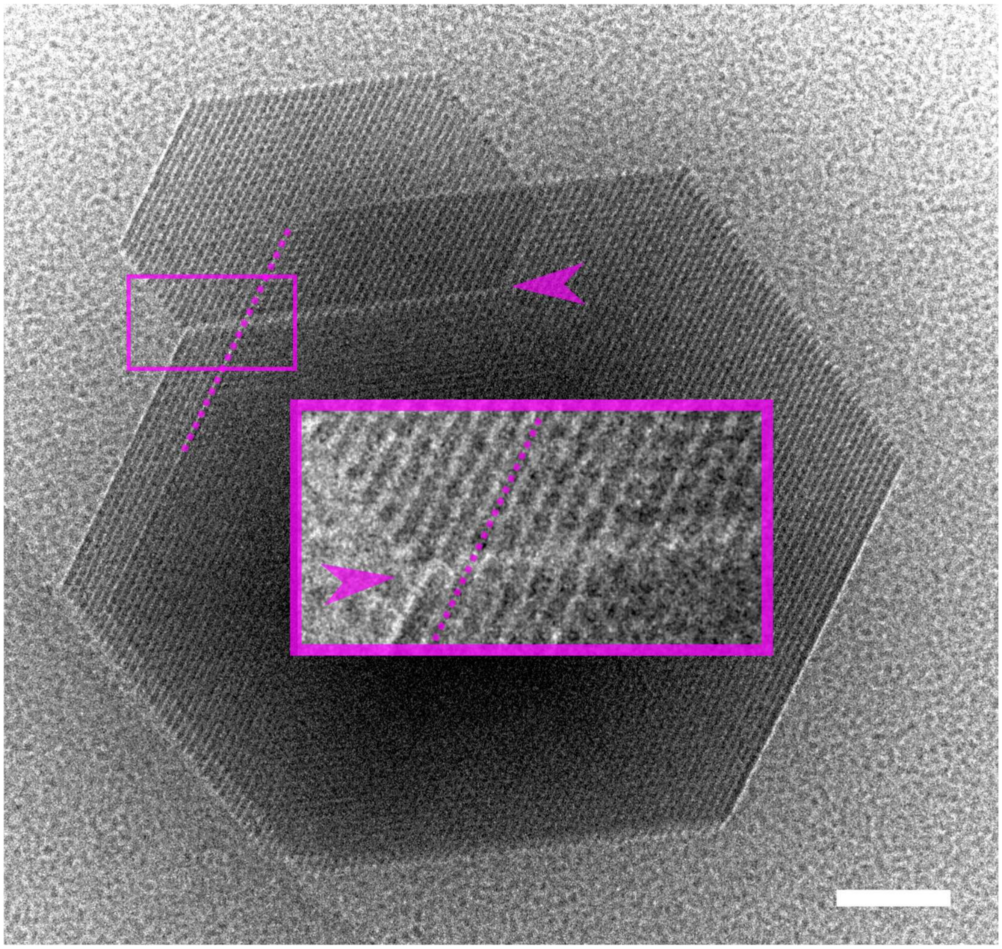

Supporting Figure 5: Three I222 GI nanocrystals in near-perfect co-alignment: the expanded window features a zoom-in of the contact region resolving a narrow solvent gap between two crystals. The dashed line highlights the alignment of the respective lattice planes of both crystals. Scale bar represents 100nm.
